# Prospective observational study of early respiratory management in preterm neonates less than 35 weeks of gestation

**DOI:** 10.1186/s12887-019-1518-3

**Published:** 2019-05-11

**Authors:** Fernando R. Moya, Jan Mazela, Paul M. Shore, Steven G. Simonson, Robert Segal, Phillip D. Simmons, Timothy J. Gregory, Carlos G. Guardia, Judy R. Varga, Neil N. Finer, on behalf of the Preterm Neonate Early Respiratory Management Prospective Observational Study investigators

**Affiliations:** 1 University of North Carolina School of Medicine, Chapel Hill, NC USA; 2 Poznan University of Medical Sciences, Poznan, Poland; 3 Windtree Therapeutics, Inc., Warrington, PA USA; 4 University of California San Diego, San Diego, CA USA

**Keywords:** Respiratory management, Preterm neonate, Continuous positive airway pressure, Endotracheal intubation, Surfactant; prospective study

## Abstract

**Background:**

Current guidelines for management of respiratory distress syndrome (RDS) recommend continuous positive airway pressure (CPAP) as the primary mode of respiratory support even in the most premature neonates, reserving endotracheal intubation (ETI) for rescue surfactant or respiratory failure. The incidence and timing of ETI in practice is poorly documented.

**Methods:**

In 27 Level III NICUs in the US (*n* = 19), Canada (*n* = 3) and Poland (*n* = 5), demographics and baseline characteristics, respiratory support modalities including timing of ETI, administration of surfactant and caffeine/other methylxanthines, and neonatal morbidities were prospectively recorded in consecutive preterm neonates following written parental consent. Infants were divided into three groups according to gestational age (GA) at birth, namely 26–28, 29–32 and 33–34 weeks. Statistical comparisons between groups were done using Chi-Square tests.

**Results:**

Of 2093 neonates (US = 1507, 254 Canada, 332 Poland), 378 (18%) were 26–28 weeks gestational age (GA), 835 (40%) were 29–32 weeks, and 880 (42%) were 33–34 weeks. Antenatal steroid use was 81% overall, and approximately 89% in neonates ≤32 weeks. RDS incidence and use of ventilatory or supplemental oxygen support were similar across all sites. CPAP was initiated in 43% of all infants, being highest in the 29–32-week group, with a lower proportion in other GA categories (*p* < 0.001). The overall rate of ETI was 74% for neonates 26–28 weeks (42% within 15 min of birth, 49% within 60 min, and 57% within 3 h), 33% for 29–32 weeks (13 16 and 21%, respectively), and 16% for 33–34 weeks (5, 6 and 8%, respectively). Overall intubation rates and timing were similar between countries in all GAs. Rates within each country varied widely, however. Across US sites, overall ETI rates in 26–28-week neonates were 30–60%, and ETI within 15 min varied from 0 to 83%. Similar within 15-min variability was seen at Polish sites (22–67%) in this GA, and within all countries for 29–32 and 33–34-week neonates.

**Conclusion:**

Despite published guidelines for management of RDS, rate and timing of ETI varies widely, apparently unrelated to severity of illness. The impact of this variability on outcome is unknown but provides opportunities for further approaches which can avoid the need for ETI.

## Background

The respiratory management of preterm infants with or at risk for respiratory distress syndrome (RDS) has evolved dramatically in neonatal intensive care units (NICUs) over the past decade. Results from several randomized trials have suggested that early use of continuous positive airway pressure (CPAP) offers potential benefits over endotracheal intubation (ETI) and mechanical ventilation (MV) with or without administration of surfactant for preterm infants [1–3]. This has led to practice guidelines and recommendations by the American Academy of Pediatrics (AAP) and other agencies to utilize CPAP as the primary mode of respiratory support even in the most premature neonates [4, 5]. A recent meta-analysis suggested that avoiding ETI and MV significantly reduces the incidence of death or bronchopulmonary dysplasia (BPD) in premature infants less than 30 weeks gestational age (GA) [6]. Furthermore, the procedure of ETI can result in complications, and primary intubation as well as reintubation have been recognized as risk factors for death and other morbidities in preterm infants [7–9].

Despite the AAP guidelines recommending CPAP as the primary mode of respiratory support even in the most premature neonates, frequently, preterm infants are intubated in the delivery room (DR) for resuscitative maneuvers and delivery of surfactant [10]. Moreover, of those who get initiated on CPAP, a variable proportion fail this therapy and ultimately are intubated. Dargaville and colleagues recently reported on a large cohort of over 19 thousand inborn infants admitted to NICU’s from the Australia and New Zealand Neonatal Network between 2007 and 2013 [11]. Infants who did not need respiratory support in the first 24 h after birth or those who had rupture of membranes for > 14 days (approximately 14% of the original cohort) were excluded. About 70% of infants between 25 and 28 completed weeks and 21% of those between 29 and 32 weeks were intubated before CPAP was initiated. Among those managed initially on CPAP, 43 and 21% of those GA groups experienced CPAP failure, respectively. Infants who failed CPAP were at higher risk for death and other adverse outcomes. The timing of CPAP failure and reasons were not described in detail.

Many single center reports published prior to the Dargaville report had suggested that the most common cause of CPAP failure among preterm infants is surfactant deficiency, probably because avoiding ETI delays the usual approach to surfactant replacement therapy [12, 13]. More recently, alternative “less invasive” or “minimally invasive” approaches for surfactant administration have been advocated such as “LISA” or “MIST”, however these are not widely used in all regions [14, 15]. Given these changes in approaches to respiratory management, it still remains unclear what proportion of preterm infants at a given GA need ETI and surfactant replacement therapy, as well as the timing and reasons for these therapeutic interventions. Thus, our objective was to prospectively identify, describe, and compare in a broad, non-selective and contemporary cohort of preterm neonates their initial respiratory management, with particular emphasis on the incidence, indications, timing and conditions resulting in ETI and surfactant administration. We sought to characterize these events in all preterm infants, without exclusions, admitted to NICU’s across several geographical areas.

## Methods

This prospective observational study was reviewed and approved by institutional review boards, and/or research ethics boards. After obtaining written informed parental/legal representative consent, we prospectively recorded pertinent data in all consecutive preterm neonates between 26 + 0 and 34 + 6 weeks GA admitted to 27 Level III NICUs in the US (19 sites), Canada (3 sites) and Poland (5 sites), see Appendix. All data collected were de-identified to ensure compliance with patient privacy rights. The information recorded included demographic and baseline characteristics, as well as pregnancy-related history including administration of antenatal steroids. In addition, we collected more detailed clinical data primarily focused on the initial use of respiratory support modalities including utilization of CPAP, timing and reasons for ETI, administration of surfactant and caffeine/other methylxanthines, and occurrence of neonatal morbidities during the first 7 days after birth. Investigators could designate more than one reason for ETI. Notably, surfactant administration was not offered as a reason for intubation in an attempt to identify and capture the clinical factors prompting the need for surfactant.

The definitions of common neonatal morbidities used were as follows: RDS, presence of clinical signs of respiratory distress and need for supplemental oxygen with chest X-Ray confirmation; patent ductus arteriosus (PDA), clinical signs and echocardiographic confirmation; intraventricular hemorrhage (IVH), seen on cranial ultrasound and graded as described by Papile et al. [16]; and necrotizing enterocolitis (NEC), presence of clinical and radiographic signs as described by Bell et al. [17].

Data were de-identified at sites and centrally collected. Infants were divided into three groups according to their GA at birth, namely 26 + 0 to 28 + 6 weeks, 29 + 0 to 32 + 6 weeks and 33 + 0 to 34 + 6 weeks. Gestational age assignment was based on last menstrual period or on Ballard assessment postnatally.

Statistical comparison between groups were done using Chi-Square tests.

## Results

From May 2015 to July 2016 a total of 2093 preterm neonates were enrolled and provided evaluable information. The number of neonates from each country were as follows: 1507 from the USA (19 NICU’s), 254 from Canada (3 NICU’s) and 332 from Poland (5 NICU’s). Of these, 378 (18%) were 26–28 weeks GA, 835 (40%) were 29–32 weeks, and 880 (42%) were 33–34 weeks. Other characteristics of this cohort are listed in Table 1. Antenatal steroid exposure was inversely related to gestational age; 81% of all infants and 89% of neonates ≤32 weeks were exposed to antenatal steroids. The use of antenatal steroids, incidence of RDS, and the utilization of ventilatory support or supplemental oxygen were similar across all countries (data not shown).

**Table 1:** Demographic characteristics by GA

	26–28 + 6 weeks (*N* = 378)	29–32 + 6 weeks (*N* = 835)	33–34 + 6 weeks (*N* = 880)	Overall (*N* = 2093)
Gestational age, mean (SD)	27.4 (0.84)	31.0 (1.18)	33.9 (0.56)	31.6 (2.50)
Male, *n* (%)	203 (54%)	449 (54%)	499 (57%)	1151 (55%)
Cesarean delivery, *n* (%)	258 (68%)	574 (69%)	534 (61%)	1366 (65%)
SGA, n (%)	43 (11%)	96 (11%)	102 (12%)	241 (12%)
Antenatal Steroids, *n* (%)	339 (90%)	746 (89%)	614 (70%)	1699 (81%)
Maternal morbidity, *n* (%)				
Chorioamnionitis	42 (11%)	37 (4%)	24 (3%)	103 (5%)
Preeclampsia/PIH	105 (28%)	273 (33%)	300 (34%)	678 (32%)
PROM	70 (19%)	151 (18%)	107 (12%)	328 (16%)

*SGA* Small for gestational age, *PIH* Pregnancy induced hypertension, *PROM* Premature rupture of membranes if diagnosed at least 48 h before birth

Overall rates of infants diagnosed with RDS and managed with non-invasive respiratory support (CPAP) are shown in Table 2, as are rates of CPAP failure and intubation. As expected, a larger proportion of neonates between 26 and 28 + 6 weeks were diagnosed with RDS compared to those groups with more advanced GA, 29 weeks and greater, whether intubated within 15 min of birth or after 15 min of birth, including those managed initially with non-invasive respiratory support (Tables 2 and 3). This was also reflected in the distribution of neonates given surfactant (Table 3). Of note, the standard approach of ETI, followed by MV remained the most common approach for surfactant administration in those NICU’s reporting data to our study. Also, the use of methylxanthine, particularly caffeine, was very common, especially among neonates < 32 weeks. Across the entire population, median times for starting caffeine/other methylxanthines were 3.5, 3.5 and 2.2 h of age for the three GA groups, respectively. Overall, 43% of all infants were started on CPAP; there is a significant difference (*p* < 0.001) when comparing he number of infants started on CPAP across GA groups (the majority of neonates in the 29 to 32-week group, but a lower proportion of the other GA categories; Table 2). Median times for starting this therapy by GA category were 0.25, 1.50 and 3.16 h, respectively. Not surprisingly, CPAP failure was higher at lower GA, as was RDS diagnosis, which were both significantly different when compared across GA groups. As expected, the incidence of RDS and surfactant use was substantially higher in infants intubated within 15 min of birth versus those not intubated before 15 min of birth, including those treated initially non-invasively (31 and 21% respectively), regardless of the gestational ages (Table 3).

**Table 2:** Respiratory Interventions by GA - All subjects

	26–28 + 6 weeks (*N* = 378)	29–32 + 6 weeks (*N* = 835)	33–34 + 6 weeks (*N* = 880)	Overall (*N* = 2093)
Diagnosed with RDS, *n* (%)	207 (55%)	330 (40%)	195 (22%)	732 (35%)
Started on CPAP^a^, *n* (%)	150 (40%)	441 (53%)	308 (35%)	899 (43%)
CPAP Failure^a^, *n/N* (%)	75/150 (50%)	114/441 (26%)	62/308 (20%)	251/899 (28%)
Endotracheal Intubation, *n* (%)	286 (76%)	278 (33%)	142 (16%)	706 (34%)

*RDS* Respiratory distress syndrome Denominator is all infants in the gestation category, unless otherwise indicated

^a^Significant between GA groups at *p* < 0.001

**Table 3:** Respiratory support for subjects intubated early (< 15 min from birth) compared with subjects managed initially with non-invasive respiratory support and/or intubated ≥15 min from birth

	Intubated < 15 min of Birth				Not intubated < 15 min of Birth			
	26–28 + 6 weeks	29–32 + 6 weeks	33–34 + 6 weeks	Overall (*N* = 310)	26–28 + 6 weeks	29–32 + 6 weeks	33–34 + 6 weeks	Overall (*N* = 1783)
	(*N* = 157)	(*N* = 111)	(*N* = 42)		(*N* = 221)	(*N* = 724)	(*N* = 838)	
Diagnosed with RDS, *n* (%)	107 (68%)	62 (56%)	18 (43%)	187 (60%)	100 (45%)	268 (37%)	177 (21%)	545 (31%)
Surfactant, *n* (%)	142 (90%)	90 (81%)	21 (50%)	253 (82%)	118 (53%)	170 (23%)	83 (10%)	371 (21%)
Standard approach	135 (95%)	82 (91%)	20 (95%)	237 (94%)	87 (74%)	105 (62%)	57 (69%)	249 (67%)
INSURE	5 (3%)	2 (2%)	0	7 (3%)	22 (19%)	53 (31%)	19 (23%)	94 (25%)
LISA	0	0	0	0	6 (5%)	8 (5%)	1 (1%)	15 (4%)
Not available, *n* (%)	2 (1%)	6 (7%)	1 (5%)	9 (4%)	3 (3%)	4 (2%)	6 (7%)	13 (4%)
Methylxanthines, *n* (%)	153 (98%)	99 (89%)	9 (21%)	261 (84%)	216 (98%)	509 (70%)	133 (16%)	858 (48%)
Caffeine, *n* (%)	147 (96%)	94 (95%)	8 (89%)	249 (95%)	174 (81%)	439 (86%)	123 (92%)	736 (86%)
Aminophylline, *n* (%)	5 (4%)	5 (5%)	1 (11%)	11 (5%)	42 (19%)	68 (13%)	9 (7%)	119 (14%)
Not Available, *n* (%)	1 (< 1%)	0	0	1 (< 1%)	0	2 (< 1%)	1 (1%)	3 (< 1%)
Started on CPAP, *n* (%)	8 (5%)	5 (5%)	1 (2%)	14 (5%)	139 (63%)	429 (59%)	307 (37%)	875 (49%)
CPAP Failure, *n* (%)	8 (100%)	5 (100%)	1 (100%)	14 (100%)	67 (48%)	109 (25%)	61 (20%)	237 (27%)
Endotracheal Intubation, *n* (%)	157 (100%)	111 (100%)	42 (100%)	310 (100%)	138 (62%)	165 (23%)	95 (11%)	398 (22%)

Category values are n (%), calculated from N for each group. Subcategory values are n (%), calculated from the category n

Note: endotracheal intubation is intubation at any time

**Fig. 1:**
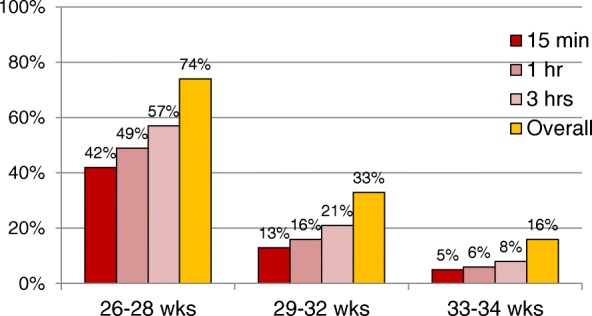
Proportion of subjects intubated by the time indicated

**Fig. 2:**
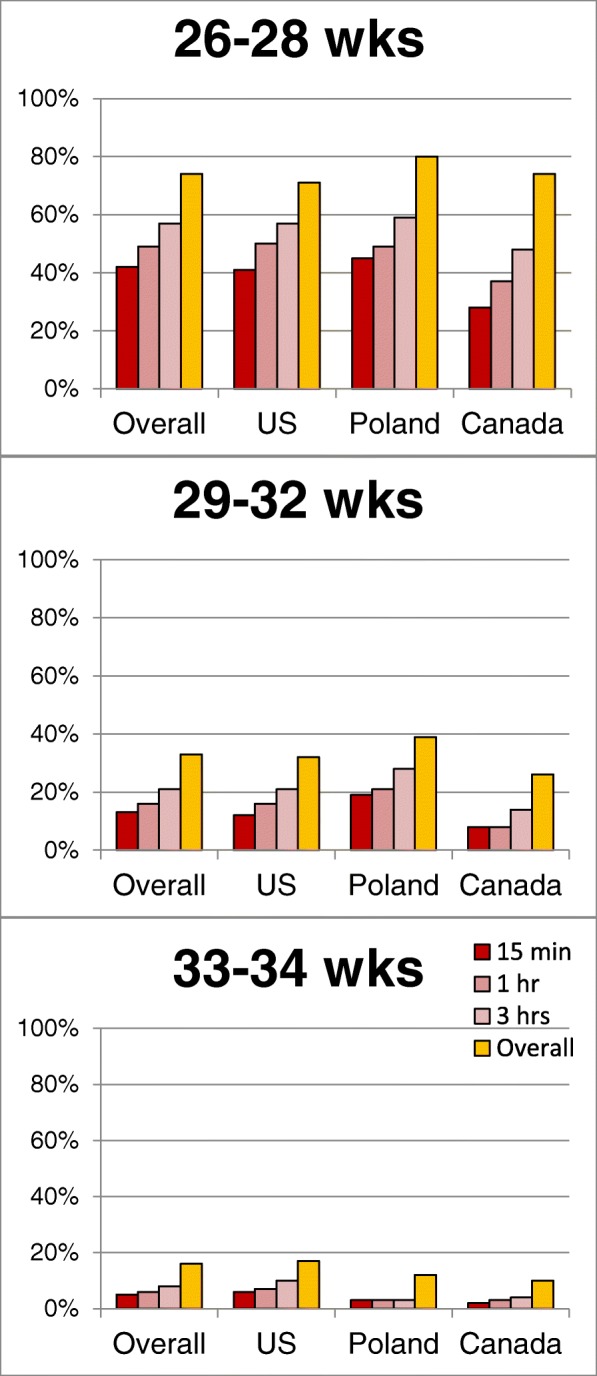
Intubation by GA and Country

**Fig. 3:**
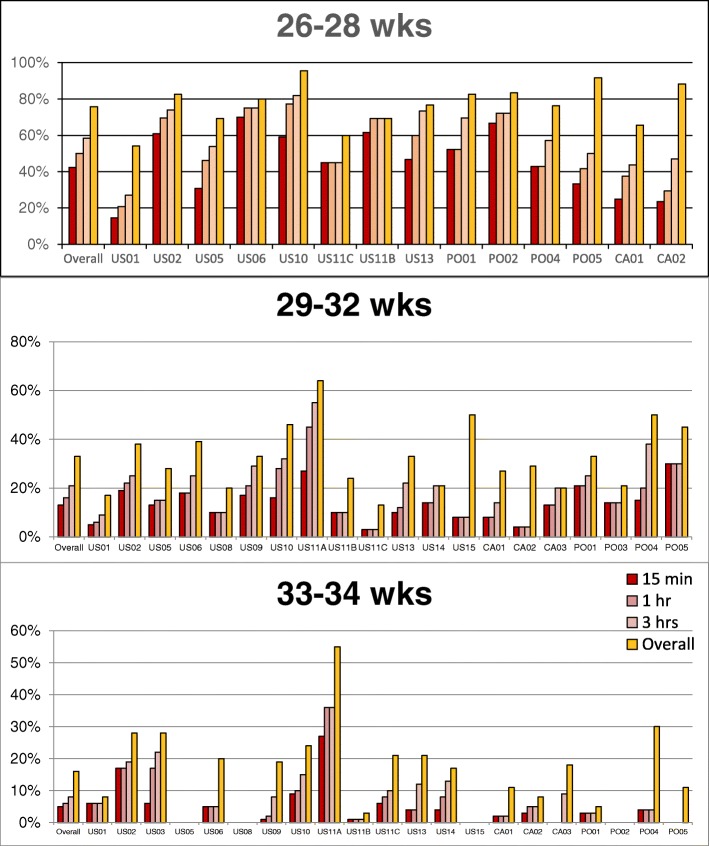
Intubation by GA and Center. Within each GA range, intubation rates across sites appear to be variable. Sites with ≥10 subjects in a GA category shown

Overall rates of ETI are shown in Fig. 1. These were approximately 74% for neonates 26–28 weeks, 33% for 29–32 weeks, and 16% for 33–24 weeks. Overall rates and timing of ETI were similar between countries in the cohorts (Fig. 2); however, rates across sites within each country varied widely (Fig. 3). Across US sites, rates of ETI in neonates 26–28 weeks within 15 min varied from 30 to 60% at most sites, but for sites that enrolled at least 5 subjects in this age group, it was as low as 0% and as high as 83%. Sites with low rates of ETI within 15 min did not necessarily have higher rates of ETI later. Similar variability was seen within Poland (71–83% overall; 22–67% within 15 min) and Canada (64–100% overall; 24–75% within 15 min) in this GA group, and within all countries for neonates between 29 and 32 and 33–34 weeks.

The most commonly stated reasons for ETI (besides “other”, which typically included need for surfactant administration) are shown in Table 4**,** broken down by those who were intubated within15 min of birth versus those who were not intubated within 15 min of birth, including those initially treated with non-invasive respiratory support. Reasons for intubation differ markedly between infants intubated before or after 15 minutes of life. Reported reasons for intubation were similar in US and Poland sites, but fewer reasons were stated in Canadian sites where MV was rarely chosen as the reason for ETI.

**Table 4:** Reasons for endotracheal intubation by GA

	Intubated < 15 min of Birth				Not intubated < 15 min of Birth			
	26–28 + 6 wks	29–32 + 6 wks	33–34 + 6 wks	Overall	26–28 + 6 wks	29–32 + 6 wks	33–34 + 6 wks	Overall
	(*N* = 157)	(*N* = 111)	(*N* = 42)	(*N* = 310)	(*N* = 221)	(*N* = 724)	(*N* = 838)	(*N* = 1783)
Airway Stabilization	25 (16%)	21 (19%)	3 (7%)	49 (16%)	13 (6%)	11 (2%)	9 (1%)	33 (2%)
Apneic Episode	36 (23%)	34 (31%)	7 (17%)	77 (25%)	25 (11%)	14 (2%)	10 (1%)	49 (3%)
Desaturation	0	0	0	0	0	0	0	0
Hypercapnia	2 (1%)	1 (1%)	0	3 (1%)	13 (6%)	21 (3%)	6 (1%)	40 (2%)
MV Administration	94 (60%)	61 (55%)	23 (55%)	178 (57%)	45 (20%)	37 (5%)	25 (3%)	107 (6%)
Increased WOB	31 (20%)	26 (23%)	2 (5%)	59 (19%)	42 (19%)	59 (8%)	33 (4%)	134 (8%)
Resuscitative Measures	103 (66%)	67 (60%)	29 (69%)	199 (64%)	23 (10%)	15 (2%)	9 (1%)	47 (3%)
FiO_2_ > Unit Threshold	20 (13%)	19 (17%)	2 (5%)	41 (13%)	53 (24%)	68 (9%)	29 (3%)	150 (8%)
Other	65 (41%)	43 (39%)	19 (45%)	117 (38%)	62 (28%)	107 (15%)	60 (7%)	229 (13%)

All values are n (%), calculated from N for each group. Multiple reasons could be checked

*Wks* weeks, *MV* Mechanical ventilation, *WOB* Work of breathing

Note: Surfactant was not provided as a reason for intubation on the collection tool (see text)

Overall mortality during the first 7 days after birth was low (Table 5). Also, air leaks were observed infrequently, and the majority were pneumothoraces (data not shown). As expected, the rate of common morbidities associated with prematurity observed during the first 7 days was higher at lower GA. No data were collected beyond 7 days of life given the study objectives of capturing data during the first 7 days of life; therefore, the incidence of complications of prematurity is undoubtedly underestimated since the entire neonatal period has not been considered.

**Table 5:** Mortality and common neonatal morbidities during the first 7 days

	26–28 + 6 weeks	29–32 + 6 weeks	33–34 + 6 weeks	Overall
	(*N* = 378)	(*N* = 835)	(*N* = 880)	(*N* = 2093)
Death	7 (2%)	4 (< 1%)	4 (< 1%)	15 (1%)
Air leaks	21 (6%)	24 (3%)	20 (2%)	65 (3%)
Acquired sepsis	27 (7%)	41 (5%)	25 (3%)	93 (4%)
PDA	42 (11%)	23 (3%)	7 (1%)	72 (3%)
IVH (all grades)	57 (15%)	56 (7%)	12 (1%)	125 (6%)
NEC (all grades)	11 (3%)	5 (1%)	4 (< 1%)	20 (1%)

All values are n (%). *PDA* Patent ductus arteriosus, *IVH* Intraventricular hemorrhage, *NEC* Necrotizing enterocolitis

*Note: the incidence of mortality and complications of prematurity was based on data collection during the first 7 days of life and not throughout the neonatal period*

## Discussion

Presently, the respiratory management of preterm infants with or at risk for respiratory problems frequently involves the use of CPAP as the first line of therapy. This has been recommended for even the most premature neonates [4, 5]. Whereas this approach may lower the risk of death and BPD, it remains unclear what proportion amongst all preterm infants born at a certain GA are actually able to be managed successfully only with CPAP, especially at lower gestational ages. Large randomized trials comparing CPAP to other approaches (e.g. ETI and surfactant administration) have focused on more selected populations because of their eligibility criteria, which usually involved more stable preterm infants not in need of resuscitation [1, 3, 10]. This hinders the generalizability of those findings to all or most preterm infants of similar gestational ages. Thus, it becomes quite important to have good estimations of what the initial respiratory management entails for all infants at a given gestational age.

Our large, contemporary data obtained across various geographic regions demonstrate that a substantial proportion of preterm infants still undergo ETI. Not unexpectedly, this occurs more often at lower GA, with the frequency of ETI essentially double for infants 26–28 + 6 weeks compared to those at 29–32 + 6 weeks and is four times higher compared to infants 33–34 + 6 weeks. Amongst those infants 26 to 28 + 6 weeks in our study, about 75% were eventually intubated, and most ETI occurred in the first three hours after delivery. A recent report by Chawla and colleagues [18] using data from the SUPPORT trial conducted by the Neonatal Network of NICHD revealed that 81% of infants enrolled between 24 and 28 weeks GA were intubated during the first 24 h after birth. Also, recently published data including infants < 28 weeks cared for in Canadian NICU’s showed that at least 74% were intubated to receive surfactant [10]. It is even possible that additional infants were intubated and then extubated without receiving surfactant. Within the Australia and New Zealand Neonatal Network, 70% of infants between 25 and 28 weeks GA and 21% of those 29–32 weeks GA were intubated [11]. Data from these reports and our own contemporary study are remarkably consistent in these findings and reveal that ETI is used frequently among preterm infants, despite recent recommendations and relatively high exposure to antenatal steroids. Our data also show that across the regions involved in our study the proportion of preterm infants that were intubated was remarkably similar. However, within each region there was substantial variability among centers. Such variability is not uncommon in clinical practice, especially over time, and has been previously shown for specific interventions and outcomes [11, 19, 20]. This notwithstanding, the overall frequency of ETI reported by most centers in our study is within what has been published in several other studies [2, 10, 11, 18, 19].

Our study attempted to establish the reasons why ETI was used as determined by participating investigators (see Appendix). In the three GA categories, the most common reasons were for resuscitative measures and the need to provide mechanical ventilation. Whereas the question of whether ETI was used specifically for administration of surfactant was not asked directly, FiO_2_ needs above their NICU threshold and increased work of breathing, a surrogate for respiratory distress, were also relatively common and followed the same progression of being more frequent at lower gestational ages. As expected, surfactant use was high in infants who were intubated early, and still frequent in those infants who were supported non-invasively initially (53, 23 and 10%, respectively in the three GA categories evaluated (Table 3). The proportion of infants diagnosed with RDS was slightly lower but followed the same pattern. This difference was greater in the lower GA category probably reflecting that some infants received surfactant prophylactically, especially if they were intubated very early. It is noteworthy that currently a significant number of preterm infants still develop RDS and receive surfactant, in spite of adequate exposure to antenatal steroids. Overall, 89% of infants ≤32 weeks in our study were exposed to antenatal steroids. This figure is similar to other recent descriptive studies or clinical trials of respiratory interventions [1–3, 10, 11, 14, 15, 18, 19].

The method used for administration of surfactant is evolving to less invasive approaches [14, 15, 21]. These techniques seek to avoid classic ETI using an endotracheal tube and MV. Albeit brief and using a different device (catheter as opposed to an ETT), this approach still requires intubation of the trachea. Many of the controlled trials of these new approaches have not been large in size and have reported variable improvements in clinical outcomes [3, 14, 22, 23]. A recent systematic review utilizing network meta-analysis suggested that “less invasive surfactant administration” (LISA), a procedure in which surfactant is administered into the lower airway after cannulation using a smaller flexible tube (like a nasogastric tube), results in less death or BPD than using other techniques [24] This notwithstanding, LISA was infrequently used among the 27 NICU’s that participated in our study and the preferred method of surfactant administration reported involved ETI. This technique requires appropriate skill and experience to visualize and insert a small catheter through the vocal cords [10, 15].

Our data indicate that slightly less than half of all infants between 26 and 34 + 6 weeks GA are started on CPAP. The GA category in which CPAP was started more frequently was between 29 to 32 + 6 weeks. The low proportion of infants that underwent a CPAP trial in the DR (i.e. first 15 min of life) reflects the need of intubation and resuscitation maneuvers in the sicker infants (high incidence of RDS regardless of GA) or a lack of willingness to provide a trial of CPAP. Dargaville and colleagues reported starting CPAP on a higher proportion of these infants, but they only reported on infants who developed respiratory distress, whereas our study included all infants [11]. This notwithstanding, as demonstrated in our findings and other reports, CPAP failure is relatively common among preterm infants, especially at lower gestational ages [11, 12]. We did not characterize the pressures used while on CPAP or which type of CPAP was utilized. Rather, we focused on why infants underwent ETI, which included those who were started on CPAP. Recently, an additional report by Dargaville and colleagues suggested that selective use of minimally invasive surfactant administration improves the success of CPAP among infants 29–32 weeks GA [15]. These methods are, as noted above, not without risk since visualization of the vocal cords and use of a laryngoscope are still required. Having alternative ways to administer surfactant without ETI should increase the likelihood of avoiding MV and potentially decreasing other morbidities. Recent, albeit few and relatively small studies, have focused on administration of surfactant without invading the lower airway, namely using a laryngeal mask airway, and, via aerosolization [25–27]. If these approaches are shown to be successful, it would provide additional tools with which to improve the respiratory management of preterm infants.

Since the publication by Schmidt and colleagues of a potentially beneficial effect of caffeine on lowering BPD among preterm infants < 1250 g at birth, its use has dramatically increased [28, 29]. In that trial, caffeine was started at a median age of 3 days. A more recent smaller trial reported improved hemodynamics and a strong trend towards lower need for intubation by administration of caffeine within 2 h after birth [30]. This preliminary finding was not substantiated by a large, observational trial by Patel et al., which did not find that early caffeine administration on the day of birth decreased the rate of CPAP failure in very low birth weight infants [29]. It should be noted that LISA and related techniques are most commonly performed after the infant has received caffeine, often within the first 30–60 min of life. Our data clearly show that caffeine/other methylxanthines are used widely and started early; however, our study did not evaluate the timing of caffeine treatment relative to ETI.

This observational study, collected data in prospective manner from all neonates admitted to the NICU who met entry criteria and for whom informed consent was obtained. Participating centers employed their own standards of practice and different approaches to managing infants across the broad GA spectrum, which could be a limitation of the study, but also reflects the reporting of “real-world” management of preterm infants 26 to 34 completed weeks GA. We did not standardize definitions for parameters such as WOB, INSURE and LISA techniques; this may account for the variability we observed across centers and regions with respect to differences in patient management. Notwithstanding, our data are consistent with those from the recent report by Beltempo and colleagues who employed a web-based survey to evaluate practice patterns across units using common unit-level practice rather than personal opinions/practice in evaluating respiratory management of extremely preterm infants [31]. This multiregional survey showed marked variations in practice in respiratory management of extremely preterm infants, as we observed, but some similarities across networks, as we saw across all regions.

## Conclusion

In conclusion, our contemporary data from 27 NICU’s enrolling over 2000 preterm infants in three countries demonstrate that preterm infants between 26 and 34 + 6 weeks GA often undergo ETI, in spite of a high rate of antenatal steroid use and frequent utilization of early CPAP and relatively early caffeine/other methylxanthines. Despite published guidelines for management of RDS, rate and timing of ETI varies widely, apparently unrelated to severity of illness and often without a trial of CPAP. The impact of this variability in practice is unknown. The need for ETI is higher at lower GA, and this intervention is often needed for resuscitation and management of respiratory distress. Our data provide a reasonable estimate of the proportion of infants that may benefit from employment of more standard evidence-based, non-invasive respiratory support approaches such as CPAP such to reduce ETI and MV.

## Appendix

Preterm Neonate Early Respiratory Management Prospective Observational Study investigators:

USA: Michael Antunes, MD, Christiana Care, Newark, DE; Venkataraman Balaraman,MD, Kapiolani Medical Center for Women & Children, Honolulu, HI; Nachammai Chinnakaruppan, MD, Lehigh Valley Hospital, Allentown, PA; Waldemr Carlo, MD, University Alabama at Birmingham Hospital for Women & Infants, Birmingham, AL; Cleary, MD Jerry, Abington Health, Abington, PA; Michael Cotton, MD, Duke University Hospital, Durham, NC; Sherry E Courtney, MD. University of Arkansas for Medical Sciences, Little Rock; Robert DiGeronimo, MD, University of Utah, Salt Lake City, UT; Fabian Eyal, MD, University of South Alabama, Mobile, AL; Anna Maria Hibbs, MD, University Hospital Case Medical – Rainbow Babies Children’s Hospital, Cleveland OH; Joseph Kaempf, MD, Providence-St. Vincent, Portland OR; Anup Katheria, MD, Sharp Mary Birch Hospital for Women & Newborn, San Diego, CA; John Ladino, MD, Atlantic Health System, Morristown, NJ; Gregory Martin, MD, Banner University Medical Center, Phoenix, AZ: Leif Nelin, MD, Nationwide Children’s Hospital, Columbus, OH, Ohio State University & Riverside Methodist Hospital, Columbus, OH; Yona Nicolau, MD, Loma Linda University, Loma Linda, CA; David M Riley, MD, Cook Children’s Medical Center, Fort Worth TX; Rebecca Rose, MD, Riley Hospital for Children, Indianapolis, IN;

CANADA: Michael Dunn MD, Sunny Brook Health Service; Toronto, ON; Ayman Abou Mehrem, Foothill Medical Centre, Calgary, AB; Georg Schmölzer, Royal Alexander;

POLAND: Janusz Gadzinowski, Poznan University of Medical Sciences, Poznan; Ewa Gulczynska, Lodz; Ryszard Lauterbach, Krakow; Piotr Korbal, Bydgoszcz; Katarrzyna Kornacka, Warsaw.

Declarations subsection - Ethics committees

USA:

1. Christiana Care Health System Institutional Review Board IRB00000480; FW A900006557.
2. Lehigh Valley Health Network’s Institutional Review Board.
3. The University of Alabama At Birmingham Institutional Review Board for Human Use FWA00005960.
4. Abington Memorial Hospital Institution Review Board.
5. Duke University Health System Institutional Review Board for Clinical Investigations FWA00009025.
6. University of Arkansas Institutional Review Board FWA000011952.
7. The University of Utah Institutional Review Board FWA00003745.
8. University of South Alabama Institutional Review Board IRB00000286; FWA00001602.
9. University Hospitals Case Medical Center Institution Review Board For Human Investigation IRB00000684, 00001691, 00008600; FWA00003937.
10. Providence Health and Science Institutional Review Board IRB00001196; FWA00001033.
11. Sharp Mary Birch Hospital for Women & Newborn Institutional Review Board IRB00000920; FWA00000084.
12. Atlantic Health System Institutional Review Board.
13. Institutional Review Board, The University of Utah IORG0000072; FWA00003745.
14. Nationwide Children’s Hospital Institutional Review Board, record of Ohio State University & Riverside Methodist Hospital FWA000002860.
15. Loma Linda University Institutional Review Board Research Protection Programs IRB00000383; FWA00006447.
16. Cook Children’s Health Care System Institutional Review Board IRB00001746; FWA00001339.
17. Indiana University Institutional Review Board IRB00000221; FWA00003566.
18. Children’s Hospital of Orange County Industry Track IRB IRB00001182; FWA00000255.
19. Western Institutional Review Board IRB00000533.
  - Kapiolani Medical Center for Women & Children, Honolulu, HI.
  - Banner – University Medical Center Phoenix, AZ.

CANADA:

1. Research Ethics Board of Sunnybrook Health Sciences Center.
2. Conjoint Health Research Ethics Board of The University of Calgary.
3. Health Research Ethics Board of Royal Alexander.

POLAND:

1. Uniwersytet Medyczny IM. Karola Marcinkowskiego w Poznzniu, Komisia Bioetyczna Przy Uniwersytecie Medycznym: Uchwala nr (resolution #) 401/15.
  - Janusz Gadzinowski, Poznan University of Medical Sciences, Poznan.
  - Ewa Gulczynska, Lodz.
  - Ryszard Lauterbach, Krakow.
  - Piotr Korbal, Bydgoszcz.
  - Katarrzyna Kornacka, Warsaw.

## Abbreviations

BPD: Bronchopulmonary dysplasia
CPAP: Continuous Positive Airway Pressure
ETI: Endotracheal intubation
FiO_2_: Fraction of inspired oxygen
GA: Gestational age
INSURE: INtubate, SUrfactant, Extubate
IVH: Intraventricular hemorrhage
LISA: Less invasive surfactant administration
MIST: Minimally invasive surfactant administration
MV: Mechanical ventilation
NEC: necrotizing enterocolitis
NICU: Neonatal intensive care unit
PDA: Patent ductus arteriosus
PIH: Pregnancy induced hypertension
PROM: Premature rupture of membranes
RDS: Respiratory distress syndrome
SGA: Small for gestational age
WOB: Work of breathing
